# Myocardial damage after prolonged accidental hypothermia: a case report

**DOI:** 10.4076/1752-1947-3-8459

**Published:** 2009-07-08

**Authors:** Eftychios Siniorakis, Spyridon Arvanitakis, Georgia Roulia, Petros Voutas, Constantinos Karidis

**Affiliations:** 1 Sotiria Chest Disease Hospital, Mesogeion Avenue, Athens, Greece

## Abstract

**Introduction:**

A case of cardiac toxicity due to prolonged hypothermia is reported.

**Case presentation:**

A 42-year-old woman of Caucasian origin presented with hypothermia after an accident. She developed atrial fibrillation and heart failure with minor electrocardiographic changes, which mimicked ischaemia. She recovered completely after one month of treatment for heart failure.

**Conclusion:**

Prolonged hypothermia, which mimicks ischaemia, may cause arrhythmias and heart failure.

## Introduction

Substantial thermopathology may ensue when the human body is exposed to extremely low temperatures [1]. Every unintentional decline in the core temperature below 35°C is considered to be accidental hypothermia, and when the effect is protracted the term of prolonged accidental hypothermia (PAHT) is used. Nuclei in the pre-optic anterior hypothalamus coordinate heat conservation. Activation of these thermostats and the cutaneous cold receptors initiate a cascade of compensatory physiologic events, the failure of which leads to the clinical and ultra-structural manifestation of PAHT.

Myocardial damage after exposure to extremely low temperatures is usually described using the general term "myocarditis". However, the effects of PAHT on the myocardium remain unclear and are mainly limited to the clinical picture of circulatory collapse and arrhythmogenesis [2]. A common sign in the electrocardiogram (ECG) is the convex elevation at the junction of the ST segment and the QRS complex, or the so-called Osborn wave [3,4].

Imaging modalities for the diagnosis of PAHT-dependent myocardial damage have not been described so far. In our patient, who had PAHT, echocardiography (ECHO) and cardiac magnetic resonance (CMR) imaging shed further light on the progress of myocardial structural damage.

## Case presentation

A previously healthy 42-year-old woman of Caucasian origin attempted to commit suicide on a winter afternoon in a hilly coastal area. Her intention was to fall to the sea from a tall rock. On her way to the rock, however, it became dark and she encountered a snow blizzard that made her disoriented and led to her subsequent fall towards a ravine. She was discovered after 18 hours. She had no history of alcohol use or substance misuse. She was not on any medication.

The patient was admitted to hospital in a semi-comatose condition, with a body core temperature of 29°C and no external injuries. She required warmed (43°C) intravenous fluid infusion, inotropic support and mechanical ventilation due to cardiocirculatory collapse (systolic blood pressure of 70 mmHg). Although cardiopulmonary bypass re-warming was proposed, the patient's relatives refused the use of any invasive technique. Her core temperature was restored after four hours of external electric warming.

**Figure 1:**
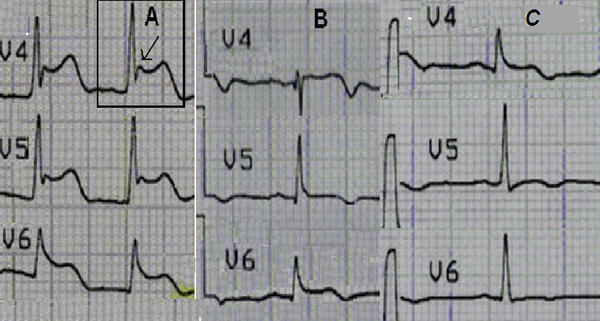
**Progressive electrocardiographic changes**. **(A)** ST segment elevations and Osborn wave (indicated by arrow) in leads V4-V6, on admission. **(B)** The same leads after re-warming. **(C)** Pattern on discharge.

An admission ECG showed atrial fibrillation with a mean rate of 85 beats per minute. The patient was administered 1 mg of atropine intravenously in the ambulance prior to admission due to bradyarrhythmia. ST segment elevation and Osborn waves were apparent in leads V4-V6 (Figure 1A). Her ECHO revealed global hypokinesia of left ventricular (LV) wall segments with an ejection fraction of 25%. A mild rise in the patient's CK (290 U/l), troponin I (4.15 ng/ml) and BNP (330 pg/ml) values was also observed. Eight hours after the patient was warmed up, her sinus rhythm was restored and Osborn waves were replaced by minor ST elevation in leads V4-V6 (Figure 1B).

**Figure 2:**
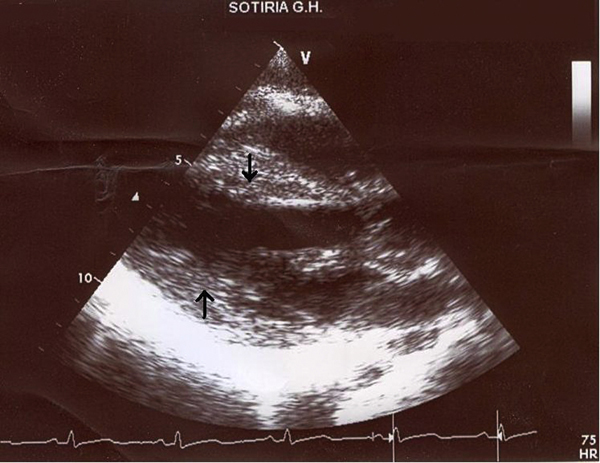
**Echocardiogram, parasternal long axis**. Arrows indicate the swelling of the intraventricular septum and the left ventricular posterior wall.

Over the next few days the patient's ECHO showed a progressive circumferential thickening of the LV wall, which is suggestive of interstitial oedema (Figure 2). Her LV contractility also showed some improvement and atypical ST changes appeared on the ECG (Figure 1C). Extubation and weaning from inotropes became possible on the third day. The patient then regained full consciousness and became asymptomatic apart from mild dyspnoea. Neurological examination was negative for focal neurological damage and a coronary angiography revealed normal coronary arteries.

**Figure 3:**
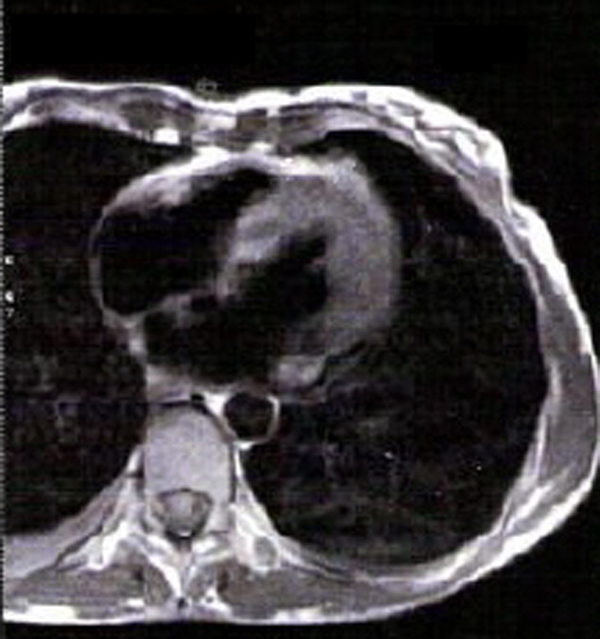
**Magnetic resonance T1 weighted image before gadolinium administration**. Hyper-enhancement of the left ventricular myocardium is seen which is compatible with inflammation and oedema.

**Figure 4:**
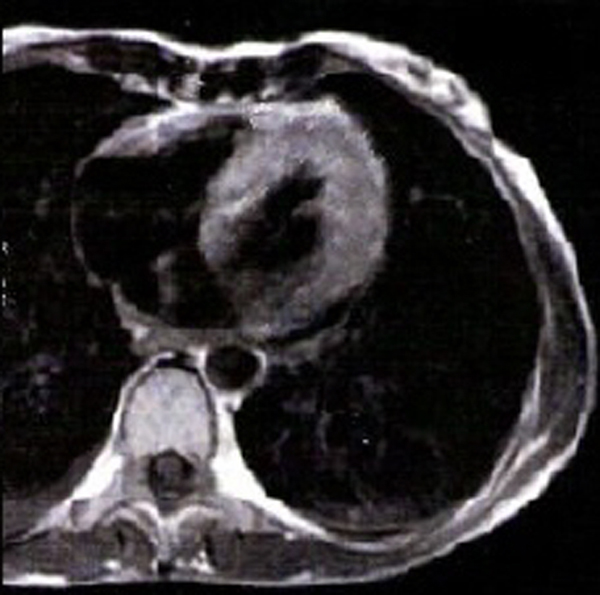
**Same image as in Figure **3**, after the administration of gadolinium**.

Concerning the ECHO findings, prolonged exposure to a cold and wet environment could have led to a viral insult on the patient's myocardium. This could account for the mild rise of troponin I and the observed ischaemia-like changes of ECG. For this reason CMR was ordered, which showed hyper-enhancement of LV myocardium on T1-weighted images, before (Figure 3) and after (Figure 4) gadolinium, compatible with diffuse myocardial inflammation and oedema. Neither focal increases of mid-wall or subendocardial signal nor residual fibrosis were found. This made the diagnosis of acute viral myocarditis, as well as the need for a CMR guided biopsy, less likely. Furthermore, a viability study excluded myocardial infarction. No other causes on which to attribute the ECHO and CMR pattern were detected apart from PAHT. Viral and immunological screenings were also negative for myocarditis.

The patient constantly improved and was discharged after 10 days. She was given ACE-inhibitors, beta-blockers and a statin. We prescribed this empirical treatment on the basis of known pleiotropic properties of such drug categories.

One month later, the patient's clinical and biochemical parameters were completely restored and there were no evidence of myocardial oedema or depressed LV functions on her ECHO. At the time of writing this case report, the patient has already completed one year of clinical and ECHO follow up examinations and is doing well without any pharmacological treatment.

## Discussion

In the setting of PAHT, various cardiovascular complications may occur [2]. In cases of mild PAHT (35°C to 32.2°C), tachycardia, bradycardia, vasoconstriction and an increase in cardiac output and blood pressure appear. Moderate PAHT (32.2°C to 28°C) as in this case provokes a progressive decrease in pulse and cardiac output and may increase atrial or ventricular arrhythmias and Osborn waves. The latter are considered to represent an epicardial-endocardial voltage gradient, which is associated with the localized appearance of a prominent epicardial action potential notch. [4] As hypothermia becomes more severe, so the appearance of Osborne waves also becomes more common. Osborn waves in this case, however, were transient and disappeared eight hours after the patient's normal temperature was restored.

Severe PAHT (<28°C) is responsible for a progressive decrease in blood pressure, heart rate and cardiac output, a burst of ventricular arrhythmias, which can terminate in asystole, and pulmonary oedema.

Pathophysiologic mechanisms involved in PAHT-induced cardiac damage comprise vasoconstriction, ventilation-perfusion mismatch, increased blood viscosity, decreased oxygen release from haemoglobin, and hypercoagulability. Epicardial petechiae, subendocardial haemorrhages and microinfarcts are found in the ventricular myocardium. This is presumably related to abnormalities in the microcirculation [5].

In this case, a mild rise in troponin I and ischaemic-like ECG alterations were observed. The patient's normal coronary angiography and CMR viability test, however, excluded any significant coronary disease.

Cardiac interstitial oedema in PAHT has not been described so far. However, myofibrillar injury and mitochondrial oedema are common and reversible ultrastructural findings in medically and experimentally induced moderate or deep hypothermic blood cardioplegia [6]-[8]. We do not know if iatrogenic hypothermia and PAHT share a common microscopic pattern of cardiac damage. In this case, ECHO and CMR detected diffuse myocardial oedema with characteristics quite different from those of myocardial infarction and/or infective myocarditis [9,10]. The latter presents a patchy distribution through the LV, which is located frequently in the lateral free wall and originates from the epicardial quartile of that wall. Other commonly seen patterns in infective myocarditis are mid-wall stria pattern in the basal interventricular septum, residual fibrosis and early changes of ventricular remodeling [11,12]. None of these patterns was found in our patient's CMR, which increased the chances that this was a PAHT induced myocardial damage. The negative results of the patient's viral and immunological tests pointed to the same conclusion.

We do not know if myocardial oedema was related to the PAHT per se or to the re-warming techniques we used. Warmed (43°C) fluid infusions and active external trunk re-warming were applied to the patient. More aggressive invasive approaches were refused by the relatives. However, several re-warming techniques are proposed for treating PAHT, including peritoneal lavage with heated crystalloid, direct hepatic rewarming, closed pleural irrigations with large bore thoracostomy tubes, closed circuit oesophageal tubes, extracorporeal arteriovenous and venovenous rewarming and haemodialysis [2,13]. All of these methods are capable of raising core temperature by 2 to 4°C per hour.

Cardiopulmonary bypass is described as the most efficient method of re-warming in PAHT. Although a rise of 1 to 2°C every five minutes was observed, when this technique was applied to PAHT victims with a core temperature of 22°C, a period of one to four hours was necessary before re-warming to 36°C. [14] The value of cardiopulmonary bypass re-warming in patients with circulatory arrest but only moderate hypothermia is doubtful. Furthermore, the upper and lower limits of the core temperature at which attempts justify the use of extracorporeal circulation remain to be fully elucidated [15].

## Conclusions

In this case, PAHT induced a core temperature of 29°C and presented with bradyarrhythmia, Osborn waves, mild troponin I increase and heart failure. Myocardial oedema detected by ECHO and further investigated by CMR, constituted an intriguing structural finding. In case this imaging pattern is validated by other observations, it can endorse a prognostic value and be used as a criterion for determining the efficacy of this re-warming technique.

## Abbreviations

CMR: cardiac magnetic resonance; ECG: electrocardiogram; ECHO: echocardiogram; LV: Left ventricle; PAHT: Prolonged accidental hypothermia.

## Consent

Written informed consent was obtained from the patient for publication of this case report and any accompanying images. A copy of the written consent is available for review by the Editor-in-chief of this journal.

## Competing interests

The authors declare that they have no competing interests.

## Authors' contributions

ES drafted the manuscript. SA helped draft and edit the manuscript. ES, GR, PV and CK looked after the patient clinically. All authors read and approved the final manuscript.
